# Tangshen formula improves diabetic nephropathy in STZ-induced diabetes rats fed with hyper-methionine by regulating the methylation status of kidney

**DOI:** 10.1186/s13148-023-01620-8

**Published:** 2024-01-02

**Authors:** Yongwei Jiang, GuoXiong Deng, Chengyin Liu, Han Tang, Jing Zheng, Xiaomu Kong, Meimei Zhao, Yi Liu, Peng Gao, Tianbao Li, Hailing Zhao, Yongtong Cao, Ping Li, Liang Ma

**Affiliations:** 1 Clinical Laboratory, China-Japan Friendship Hospital, No. 2 Yinghua East Street, Chaoyang District, Beijing, 100029 China; 2 BioChain (Beijing) Science and Technology Inc., No. 18 Hongda South Road, BDA, Beijing, 100176 China; 3 Beijing Key Lab Immune-Mediated Inflammatory Diseases, Institute of Clinical Medical Science, China-Japan Friendship Hospital, No. 2 Yinghua East Street, Chaoyang District, Beijing, 100029 China

**Keywords:** Hyper-methionine, Diabetic nephropathy, Differential methylation profiles, Tangshen formula, Methylation patterns

## Abstract

**Background:**

The objective of this study was to examine and analyze differential methylation profiles in order to investigate the influence of hyper-methioninemia (HM) on the development of diabetic nephropathy (DN). Male Wistar rats, aged eight weeks and weighing 250–300 g, were randomly assigned into four groups: a control group (Healthy, *n* = 8), streptozocin-induced rats (STZ group, *n* = 8), HM + STZ group (*n* = 8), and the Tangshen Formula (TSF) treatment group (TSF group, *n* = 8). Blood glucose levels and other metabolic indicators were monitored before treatment and at four-week intervals until 12 weeks. Total DNA was extracted from the aforementioned groups, and DNA methylation landscapes were analyzed via reduced representative bisulfite sequencing.

**Results:**

Both the STZ group and HM + STZ group exhibited increased blood glucose levels and urinary albumin/creatinine ratios in comparison with the control group. Notably, the HM + STZ group exhibited a markedly elevated urinary albumin/creatinine ratio (411.90 ± 88.86 mg/g) compared to the STZ group (238.41 ± 62.52 mg/g). TSF-treated rats demonstrated substantial reductions in both blood glucose levels and urinary albumin/creatinine ratios in comparison with the HM + STZ group. In-depth analysis of DNA methylation profiles revealed 797 genes with potential therapeutic effects related to TSF, among which approximately 2.3% had been previously reported as homologous genes.

**Conclusion:**

While HM exacerbates DN through altered methylation patterns at specific CpG sites, TSF holds promise as a viable treatment for DN by restoring abnormal methylation levels. The identification of specific genes provides valuable insights into the underlying mechanisms of DN pathogenesis and offers potential therapeutic targets for further investigation.

## Introduction

Diabetic nephropathy (DN) is a prevalent and chronic complication of diabetes. As a major risk factor for end-stage renal disease [1], DN contributes to diabetic mortality and imposes a significant burden on global healthcare systems. The pathogenesis of DN involves complex interactions among inflammatory, metabolic, and hemodynamic factors [2]. These factors can lead to injuries to the glomeruli and associated cells. Fundamental pathological changes in DN induce thickening of the glomerular basement membrane, tubulointerstitial fibrosis, the accumulation of extracellular matrix proteins, mesangial dilation, and eventual complete glomerular sclerosis [3].

DNA methylation is a significant epigenetic alteration that has a vital function in controlling gene regulation [4]. It mainly adds a methyl group to the DNA molecule on the cytosine residue of CpG dinucleotide [5]. The patterns of DNA methylation can impact gene expression by altering the accessibility of transcription factors and other regulatory proteins to the DNA sequence [6]. Recent research indicates that changes in DNA methylation may be related to the development and advancement of DN. Alterations in DNA methylation patterns can impact the expression of genes associated with renal function, inflammation, oxidative stress, and fibrosis [7].

Research has identified notable disparities in the methylation levels of fibrosis-related genes in the whole genome DNA of renal tubular cells between patients with DN and chronic renal failure and normal individuals, indicating that DNA methylation is related to the development of renal tissue fibrosis [8]. Furthermore, recent advances have highlighted the key roles of epigenetic modifications, including DNA methylation, in the pathogenesis of DN. Studies have reported that low methylation of the promoter region of IL-1β, TNF-α, and IL-17 in patients with DN may be directly associated with inflammatory levels in renal tissue [9].

Homocysteine (Hcy) is an intermediate product in methionine and cysteine metabolism, synthesized through elimination of terminal methyl groups from methionine [10]. Hcy is involved in two main metabolic pathways: (1) re-methylation to form methionine using vitamin B12 as a coenzyme, catalyzed by methionine synthase and N5, N10 methylenetetrahydrofolate reductase, and (2) synthesis of cysteine sulfide using vitamin B6 as a coenzyme, facilitated by cysteine sulfide β synthase [11]. In normal adults, the generation and clearance of Hcy maintain a strict “dynamic balance,” with a plasma concentration of approximately 5–15 µmol/L. The symptom of hyperhomocysteinemia (Hhcy) occurs when the peripheral blood Hcy levels exceeds 15 μmol/L [12]. Excessive intake of methionine or disorders in homocysteine metabolism-related factors, such as pyridoxine, folic acid, and vitamin B12, can lead to homocysteine (Hcy) accumulation and subsequently cause hyperhomocysteinemia (Hhcy) [13]. To induce Hhcy in rodents for research purposes, a hyper-methionine diet has frequently been employed [14].

The concentration of Hcy has the potential to influence the overall DNA methylation level of the entire genome, and the association between Hcy level and DNA methylation may be linked to the metabolic equilibrium of the folate cycle pathway in the methionine cycle. Firstly, in mammals, S-adenosylmethionine (SAM) serves as a methyl donor for methylation reactions, and the concentration of SAM and its reaction product, S-adenosylhomocysteine (SAH) can regulate DNA methylation reactions [15, 16]. Furthermore, SAH exhibits a strong binding affinity to the catalytic region of DNMTs, effectively functioning as a potent competitive methyltransferase inhibitor. Hence, the rapid removal of SAH is imperative for the efficacy of methylation reactions [17]. In the process of Hcy production, the hydrolysis of SAH to Hcy is a reversible reaction, and the synthesis of SAH is much more efficient compared to hydrolysis. Therefore, the rapid metabolism of Hcy is also crucial for the hydrolysis of SAH. Hcy accumulation can cause SAH accumulation, which in turn leads to inhibition of methylation reactions and a decrease in DNA methylation levels [18]. Therefore, the current view suggests that Hcy forms connections with DNA methylation through SAM and SAH in the methionine cycle, occupying an important junction between carbon metabolism and epigenetic modifications [19]. Multiple studies have demonstrated a correlation between elevated Hcy levels and reduced DNA methylation levels. In the Hhcy mouse model, the overall DNA showed a hypo-methylation state [18]. The association between Hhcy and DNA methylation is also reflected in the risk factors of cardiovascular diseases such as diabetes. In one study, 85 patients with diabetes and 30 healthy subjects were selected as controls. The results showed that compared with normal group, simple diabetes group, early or clinical diabetic nephropathy groups all have higher Hcy levels and lower methylation states [20].

Most studies suggest that the presence of Hhcy in the circulation of diabetes patients is a potent driving force for the occurrence and progression of chronic complications of diabetes [21, 22]. Elevated levels of Hcy may be linked to a reduction in the glomerular filtration rate (GFR) among individuals suffering from diabetes and diabetic nephropathy. Due to its role in the clearance and metabolism of Hcy [23], a reduction in the GFR can lead to accumulation of Hcy in the body. Thus, Hhcy may serve as a significant contributing factor to the occurrence and progression of DN, although its related mechanism remains unclear.

Tangshen Formula (TSF), a herbal medicine based on traditional Chinese medicine (TCM) theory, has been used to treat DN. Clinical trials (multicenter, double-blind, and placebo-controlled) have confirmed that TSF can reduce urinary protein levels and enhance the estimated glomerular filtration rate (eGFR) in DN patients with macroalbuminuria [24, 25]. In diabetic animal models, TSF has demonstrated promising effects in attenuating renal pathological changes in DN [26].

Based on the above, we hypothesized that elevated levels of homocysteine (Hhcy) induced by HM can aggravate the progression of DN by regulating methylation levels in animals, and TSF can treat DN by restoring specific methylation patterns. Furthermore, DNA methylation profiling has the potential to be used as biomarkers for early detection, prognosis, and response to DN treatment. Analyzing the methylation status of particular genes or genomic regions may provide insights into underlying molecular mechanisms and identify novel therapeutic targets.

## Materials and method

### Animal modeling

The animal experiments were carried out following the ARRIVE guidelines [27] and the National Institutes of Health’s guide for the care and use of laboratory animals [28]. Randomization was implemented to mitigate bias in this study. Diabetes was induced by injecting 30 mg/kg of 1% streptozocin into the peritoneal cavity, twice within a one-week period. A caudal vein blood glucose level greater than 16.7 mmol/L for three consecutive days following the second STZ injection was deemed as indicative of a standard diabetic model. The diabetic rats were divided into three groups using random assignment: the STZ group (*n* = 8, received normal feed and no treatment with distilled water gastric lavage), the hyper-methionine (HM) + STZ group (*n* = 8, received 2% hyper-methionine feed [29] and no treatment with distilled water gastric lavage), and the TSF group (*n* = 8, received 2% hyper-methionine feed and 3.60 g/kg/day of Tangshen Formula Granules through gastric lavage). Healthy Wistar rats of equivalent age were the control group. The HM + STZ group and TSF group were administered a 2% methionine (wt/wt) diet, which was dissolved in regular rat feed, for a duration of 12 weeks. Both the rats in the normal group and the STZ group were provided with standard rat diet. Tangshen Formula is made into granules by Jiangyin Tianjiang Pharmaceutical Co., Ltd (Jiangsu Province, China). Firstly, the seven herbs present in Tangshen Formula are simmered in water, and then the water extracts of each herb undergo lyophilization, resulting in the production of freeze-dried powder in a predetermined ratio. Dissolve TSF particles in distilled water at a dosage of 3.60 g/kg/day. The experimental cycle starts from the first day of administration to 12 weeks, and body weight is measured weekly to adjust volume of gastric lavage.

### Function detection

Measurements of blood glucose and triglyceride (TG) concentrations were taken prior to treatment and at four-week intervals until 12 weeks. Urine samples from rats were collected at 24-h intervals during weeks 0, 4, 8, and 12. The urinary albumin/creatinine ratio was determined by measuring the levels of albumin and creatinine in the urine.

### Histopathological stains

Following a 12-week treatment period, kidney tissue samples from rats in each group were collected for histopathological staining to observe changes in renal tissue morphology. The dissected kidney tissues were preserved in 10% neutral buffered formalin for 48 h prior to being prepared and encased in paraffin. The H&E staining technique was applied to every paraffin-embedded sample, which was then divided into slices with 3-µm in thickness. Furthermore, kidney slices were subjected to staining using periodic acid-Schiff (PAS) reagent and Masson reagent.

### Methylation analysis in renal tissue

#### Library preparation for sequencing

The first step was to extract total DNA from renal tissues, with three rats in each group. The quantity of DNA was determined by measuring A260/280 ratios, with optimal values between 1.8 and 2.0 for subsequent analysis. The DNA was fragmented using *MspI* digestion (NEB, USA) in order to prepare reduced representation bisulfite sequencing (RRBS). Following that, bisulfite conversion was conducted on the fragmented DNA samples. The adapter was subsequently affixed to individual single-stranded DNA fragments. The library preparation process encompassed multiple stages, including the Adaptase stage, the extension stage, and the ligation stage. A PCR amplification was performed to incorporate complete adapters and enhance the output. Ultimately, the sequence was performed on an Illumina Hiseq 4000 platform.

#### Bioinformatics analysis

Original reads were processed using software tools, such as Cutadapt and in-house Perl scripts. Afterward, the quality of the sequencing data was evaluated using FastQC. Bismark was employed to compare bisulfite-treated reads with the reference genome and detect methylation sites. Samtools was used to eliminate duplicate reads after the alignment process. The DNA methylation level for each cytosine site in the reference genome sequence was determined by calculating the ratio of reads supporting methylation to the total number of reads. The analysis was conducted utilizing proprietary Perl scripts and MethPipe software. The identification of differentially methylated regions (DMRs) was performed using the R package MethylKit, employing default parameters such as 1,000 bp sliding windows, 500 bp overlap, and a significance threshold of *p* value < 0.05. The data were analyzed using the software tool SPSS 22.0.

## Results

**Fig. 1:**
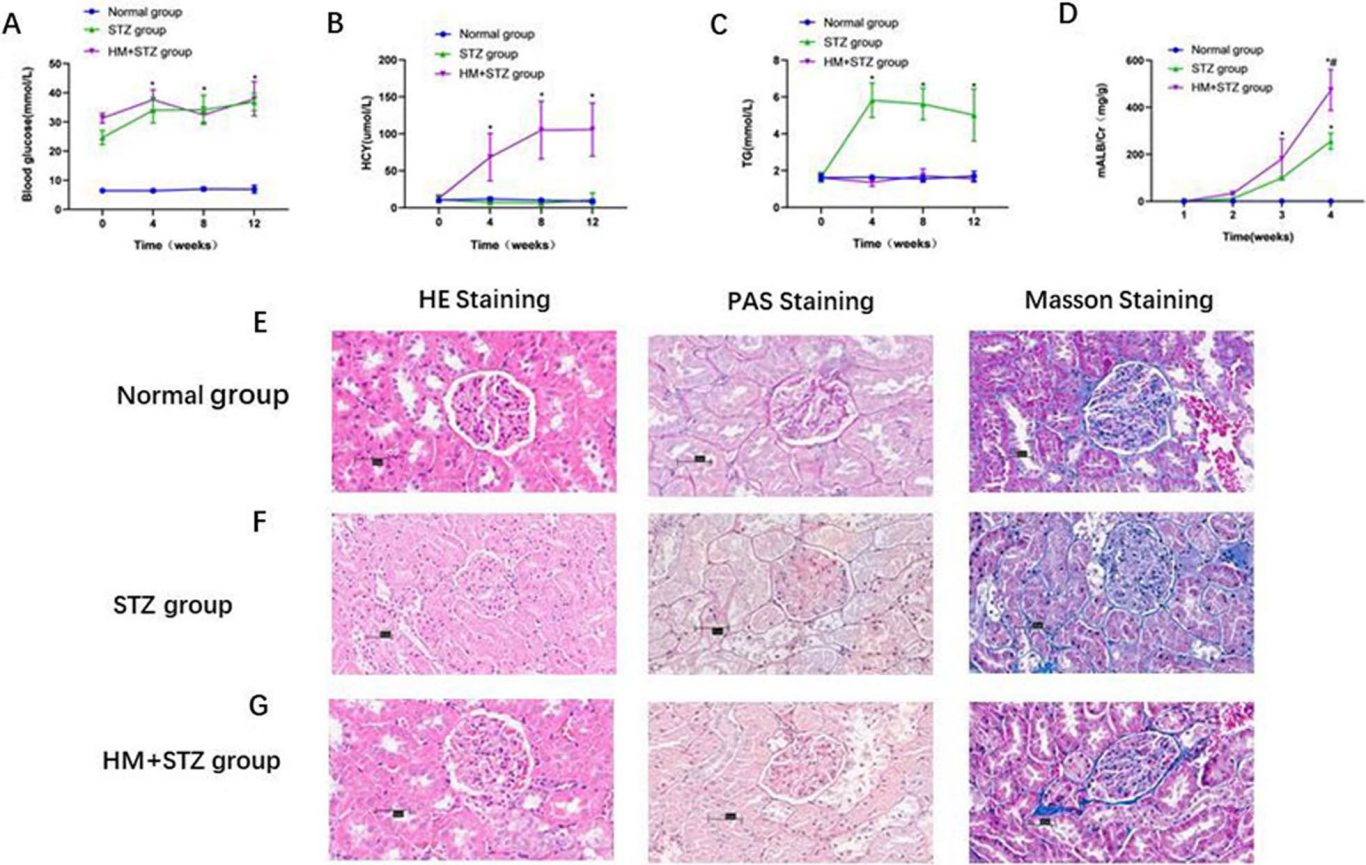
Effects of hyper-methionine (HM) diet in STZ-induced diabetic Wistar rats during 12 weeks. **A** Changes in blood glucose levels. **B** Changes in homocysteine (Hcy) levels. (**C**) Changes in triglycerides (TG) levels. **D** Changes in the ratio of mALB to Cr. **E**–**G** Glomerular morphology in diabetic rats induced by STZ was examined using HE, PAS and Masson staining after 12 weeks of either a normal diet or HM diet (At a magnification of × 400). *P < 0.05 compared to normal group. #*P* < 0.05 compared to STZ group. *n* = 8 per group. All data are expressed as mean ± SD

The blood glucose levels of both the STZ group and HM + STZ group exhibited a significantly increase compared to the control group (*P* < 0.05). Nevertheless, during the 12-week period of HM feeding, blood glucose levels were not substantial elevation in the HM + STZ group in comparison with the STZ group (Fig. 1A). The administration of STZ does not have a significant impact on the serum Hcy level when compared to the control group (*P* > 0.05). As anticipated, the serum homocysteine (Hcy) levels in HM + STZ group showed a remarkable increase starting from 4 weeks after being fed with 2% HM. Since the fourth week of HM feeding, the levels of Hcy in the HM + STZ group were 68.38 ± 30.66 μmol/L, 104.99 ± 36.43 μmol/L, and 105.69 ± 33.81 μmol/L, respectively, which were significantly higher than those of the STZ group (*P* < 0.05, Fig. 1B).

Starting from week 4, the triglyceride (TG) levels in the STZ group were significantly higher than those in the control group (5.82 ± 0.94 mmol/L, 5.62 ± 0.85 mmol/L, and 5.02 ± 1.42 mmol/L, respectively, vs. 1.64 ± 1.03 mmol/L, 1.56 ± 0.15 mmol/L, and 1.70 ± 0.26 mmol/L, respectively). After being fed a 2% hyper-methionine diet, the elevated TG levels significantly decreased. At the 4th, 8th, and 12th weeks, the TG levels in the HM + STZ group were 1.36 ± 0.20 mmol/L, 1.73 ± 0.36 mmol/L, and 1.55 ± 0.17 mmol/L, respectively, which were significantly lower than those observed in the STZ group (*P* < 0.05, Fig. 1C).

Regarding urinary albumin/creatinine levels, both the STZ group (11.80 ± 0.91 mg/g, 99.87 ± 7.82 mg/g, 255.72 ± 33.79 mg/g) and HM + STZ group (33.16 ± 6.09 mg/g, 180.83 ± 85.42 mg/g, 473.62 ± 86.99 mg/g) showed a significant increase (*P* < 0.05) from the 4th week onwards compared to the control group. When comparing urinary albumin/creatinine levels between the STZ group and HM + STZ group, at week 12, the HM + STZ group had significantly higher urinary albumin/creatinine levels (411.90 ± 88.86 mg/g) than the STZ group (238.41 ± 62.52 mg/g, *P* < 0.05, Fig. 1D). Our findings suggest that, compared to normal feeding of STZ rats, HM feeding significantly increased urinary albumin/creatinine levels in STZ rats which was consistent with the pathological changes observed in renal tissue, such as thickening of the glomerular basement membrane and glomerulosclerosis (shown in Fig. 1F, G).

**Fig. 2:**
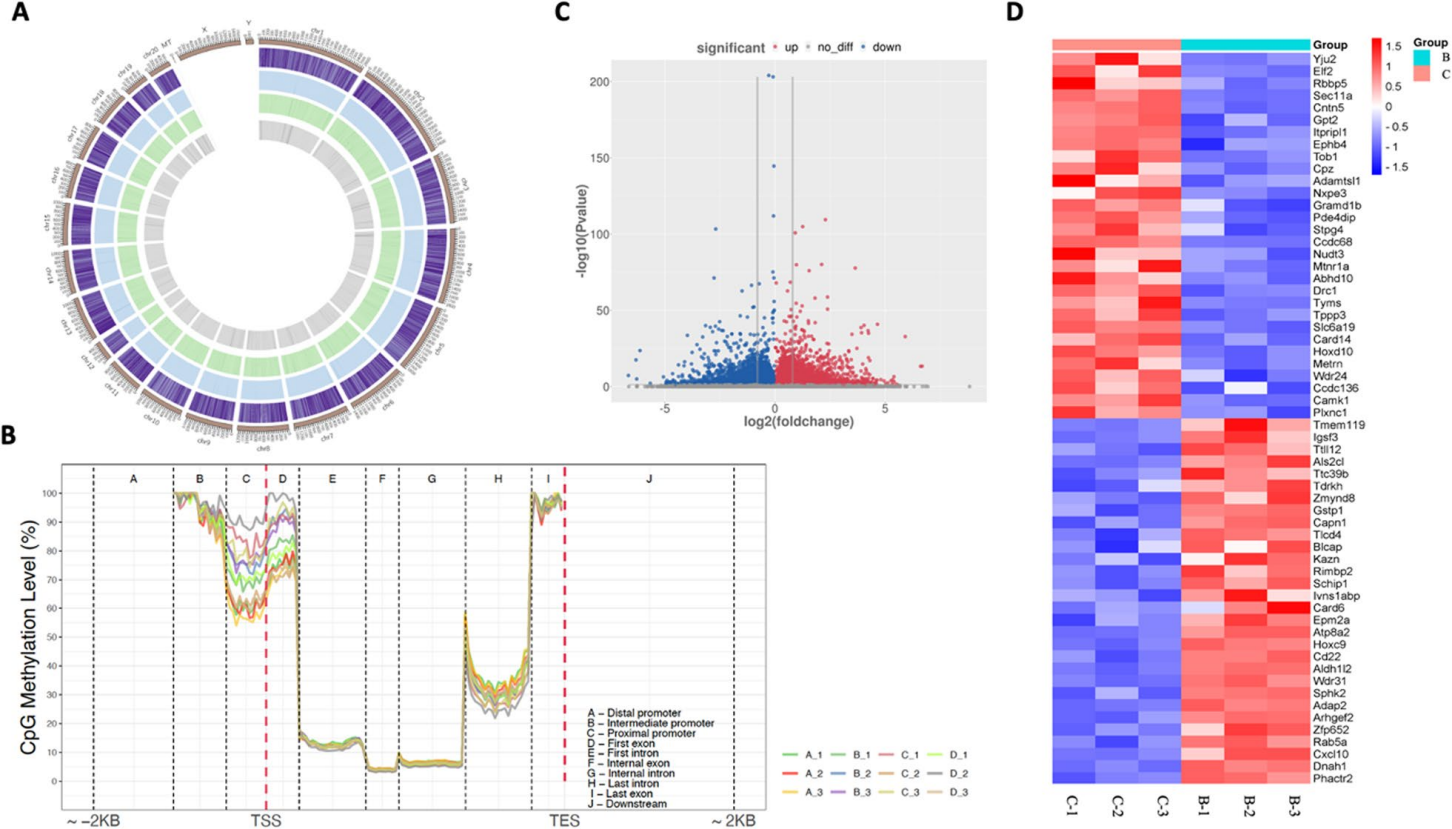
DNA methylation investigation of the aberrant DNA methylation profile in DN. **A** The circos-plot shows the overall methylation status in the whole genome; **B** Comparison of CpG methylation levels among different groups, including healthy control (Group A), STZ (Group B), HM + STZ (Group C), and TSF (Group D); **C** The volcano plot shows up-regulated and down-regulated differentially methylated genes, where the blue area stands for hypo-methylation and the red area indicates hyper-methylation; **D** The heat map illustrates the top 60 differentially methylated genes between HM + STZ group and STZ Group. The color intensity states the degree of difference in methylated genes, where darker colors represent more pronounced differences. The blue color indicates an overall status of hypo-methylation, and the red color suggests hyper-methylation

*DNA methylation analysis was conducted to* investigate the aberrant DNA methylation profile in DN. The DNA samples of the rat renal tissues from different groups, including healthy controls, STZ group, HM + STZ group, and TSF group, respectively, were sequenced by reduced representation bisulfite sequencing (RRBS) method. For each sample, we obtained more than 33.64 million raw reads with a Q30 score of over 87.54%. The mapping rate ranged from 65.29 to 76.82%, and the bisulfite conversion rate was over 99.52%. The average percentage of methylated CpG sites in each group was found to be 58.67%, 53.25%, 54.70%, and 52.86%, respectively. The genome-wide methylation profiles of the different groups were mapped and depicted in Fig. 2A. The overall methylation level did not exhibit significant changes, with only 0.22%, 0.23%, 0.22%, and 0.24% of mCpG sites showing differential methylation among the different groups. In the gene level, the most distinctive differences were noticed in the proximal promoter and the first exons, while the regions among the first intron, internal exon and the internal intron were observed with no significant difference (Fig. 2B).

To investigate the molecular alterations of hyperhomocysteinemia (Hhcy) in diabetes by comparing the HM + STZ group with the STZ group, a total of 71,957 DMRs were discovered. Among these, 48,950 (68.02%) DMRs exhibited hyper-methylation, while 23,007 (31.98%) showed hypo-methylation (Fig. 2C). The top 60 most differential methylated genes (DMGs) with a fold change over 5.78 were illustrated. Notably, genes such as *Yju2, Elf2, Rbbp5, Sec11a, Cntn5,* and *Gpt2* were found to be hypo-methylated in the STZ group but hyper-methylated in the HM-induced rats. Conversely, genes such as *Zfp652, Rab5a, Cxcl10, Dnah1,* and *Phactr2* displayed an opposite pattern of methylation status (Fig. 2D).

**Fig. 3:**
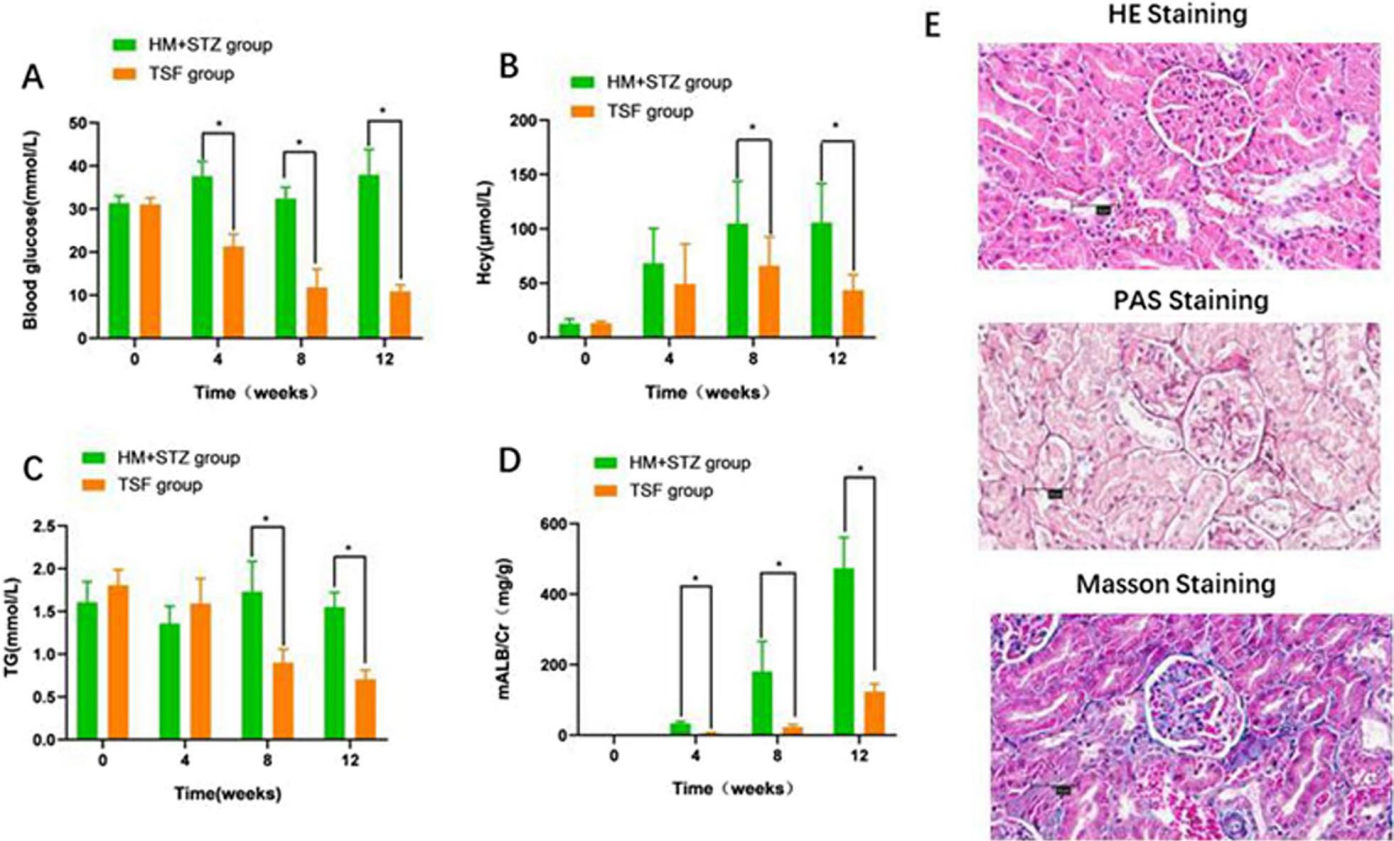
Treatment effects of Tangshen Formula in HM + STZ rats during 12 weeks. **A** Changes in Blood glucose. **B** Changes in serum Hcy. **C** Changes in triglycerides (TG). **D** Changes in mALB /Cr. **E** Glomerular morphology in TSF group stained with HE, PAS and Masson after 12 weeks treatment (× 400). *P < 0.05 compared to HM + STZ group. *n* = 8 per group. All data are expressed as mean ± SD

After 4, 8, and 12 weeks of treatment with Tangshen Formula (TSF), the blood glucose levels in the TSF group (21.32 ± 2.82 mmol/L, 11.85 ± 4.18 mmol/L, and 10.90 ± 1.49 mmol/L, respectively) were significantly reduced compared to the HM + STZ group (37.61 ± 3.44 mmol/L, 32.42 ± 2.59 mmol/L, and 37.95 ± 5.85 mmol/L, *P* < 0.05, Fig. 3A). TSF treatment can not only effectively reduce the blood glucose of diabetes rats, but also decrease the level of serum Hcy. During the study, we found that after 8 weeks of TSF treatment, the Hcy of rats in TSF group (63.37 μmol/L ± 29.47 μmol/L, 40.44 μmol/L ± 15.80 μmol/) was significantly lower than that in HM + STZ group (*P* < 0.05, Fig. 3B). Despite the effective reduction of triglyceride (TG) levels in the HM + STZ group through the 2% methionine diet, treatment with TSF still improved TG levels after 8 weeks (0.90 ± 0.16 mmol/L, *P* < 0.05) and 12 weeks (0.71 ± 0.11 mmol/L, *P* < 0.05, shown in Fig. 3C). In comparison with the HM + STZ group, the TSF group exhibited significantly lower urinary albumin/creatinine levels (5.42 ± 1.61 mg/g, 22.56 ± 7.57 mg/g, and 123.78 ± 21.51 mg/g from the 4-week to 12-week treatment of TSF, *P* < 0.05, Fig. 3D), which was consistent with the improvement of glomerulosclerosis in renal tissue (Fig. 3E). These findings demonstrate that TSF not only reduces blood glucose and triglycerides but also improves the renal function and structure in diabetic rats.

**Fig. 4:**
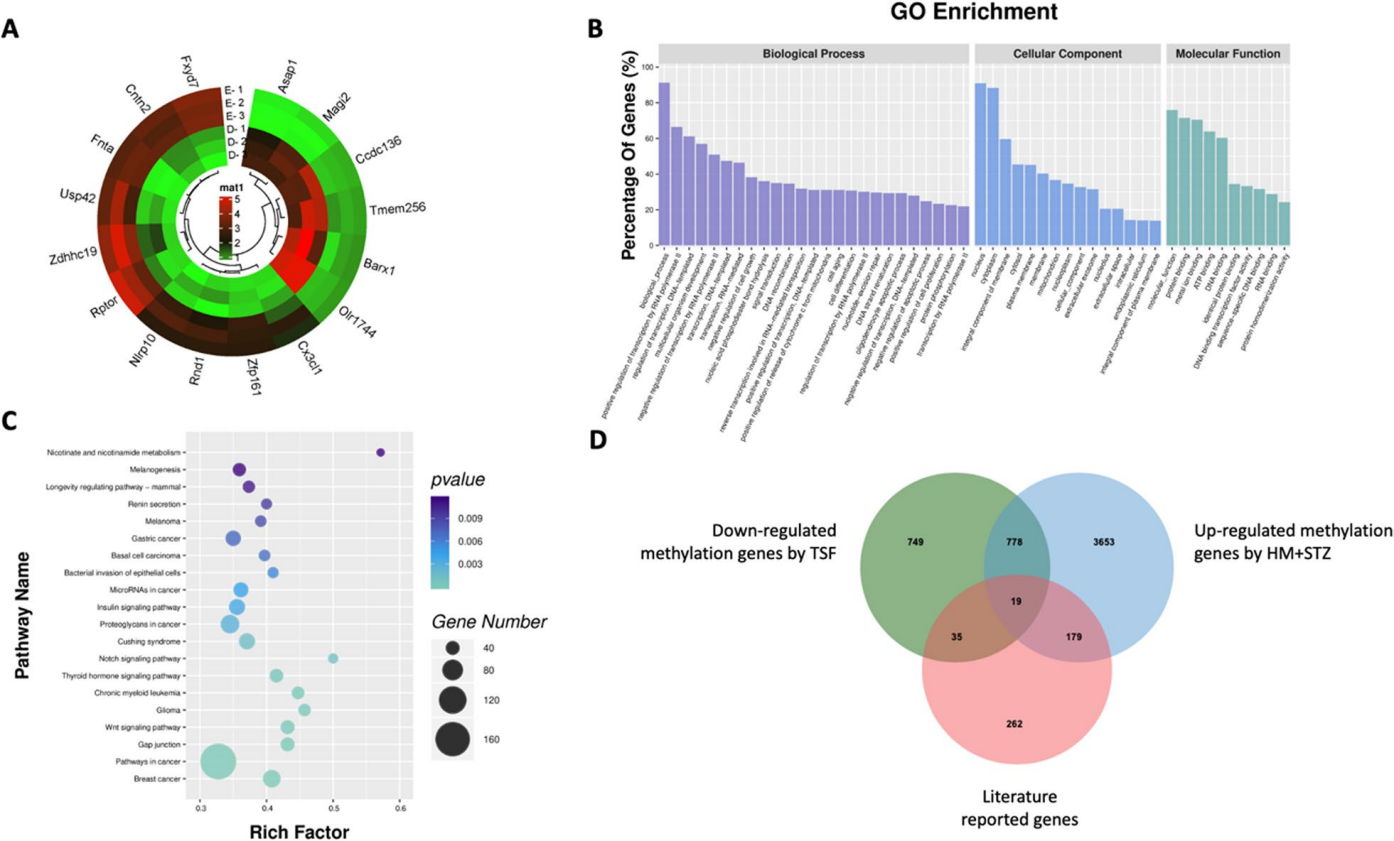
Changes in the DNA Methylation Profile of the TSF Group. **A** The circus plot indicates the changes in the methylation status of specific methylation sites. The red region represents hyper-methylation, whereas the green region represents hypo-methylation; **B** Gene Ontology (GO) analysis illustrates a large proportion of DMGs (> 60%) involved in transcription regulation and molecular binding (protein, metal-ion, ATP, and DNA) processes; **C** The Bubble plot shows the associated signaling pathways enriched in highly methylated genes; **D** The overlap of the reported and research-wised methylation-related homologous genes. The green circle indicates down-regulated methylation genes by the TSF group versus the HM + STZ group. The blue circle stands for up-regulated methylation genes by the HM + STZ group versus the control. The pink circle represents the published methylation-related homologous genes associated with homocysteine

To explore the molecular mechanism of the therapeutic effect of TSF on HM-induced diabetic rats, DNA methylation profiles between the HM + STZ and the TSF groups were compared. A total of 4,522 differentially methylated genes were identified based on methylation status change, including 3,472 down-regulated and 1,050 up-regulated genes influenced by TSF treatment. Several typical genes such as *Asap1*, *Magi2*, and *Ccdc136* exhibited hyper-methylation, whereas *Fxyd7*, *Cntn2*, *Fnta*, and *Usp42* displayed the opposite methylation changes (Fig. 4A).

Gene Ontology (GO) analysis revealed that a significant proportion of the DMGs (> 60%) were involved in biological processes, especially in both positive and negative transcription regulations by RNA polymerase II. These DMGs were approximately equally distributed between the nucleus and cytoplasm and were more pronounced in molecular function, including protein binding and metal-ion binding (Fig. 4B). The DMGs with rich factors over 0.5 and *P* value less than 0.01 were primarily enriched in the Nicotinate and nicotinamide metabolic pathway as well as the Notch signaling pathway (Fig. 4C).

Furthermore, investigating the DMGs rescued by the TSF treatment, the up-regulated DMGs between the HM-induced rats and the healthy controls were compared with the down-regulated counterparts between the TSF-treated and the TSF-untreated diabetic rats. A total of 797 genes were discovered with potential therapeutic effect related to TSF, among which about 2.3% homologous genes were previously reported. These genes with most distinctive differential methylation status, such as Mas1l, Sdk1, Duox2, Supt20h, Card10*,* KcnJ8*,* and Gstt2, revealed connections to the TSF molecular mechanism (Fig. 4D).

## Discussion

Hyperhomocysteinemia (Hhcy) is a medical condition characterized by abnormally high levels of homocysteine in the bloodstream, which has been shown in multiple studies [30–32] to be a risk factor for the onset and advancement of DN, a prevalent cause of end-stage renal disease globally. Heightened concentrations of homocysteine lead to increased oxidative stress, inflammation, impairment of the endothelium, and damage to the glomerular filtration barrier. These factors contribute to proteinuria, a hallmark of DN, as well as other diabetes complications, including retinopathy and neuropathy.

The precise mechanism linking Hhcy and DN remains incompletely elucidated. However, it has been suggested that homocysteine may induce renal injury through various pathways, such as triggering the renin-angiotensin system (RAS), increasing the production of growth factors, and forming advanced glycation end-products (AGEs). Various strategies have been proposed to mitigate the risk of Hhcy-associated DN, including dietary changes, physical activity, smoking cessation, and pharmacological approaches involving folic acid supplementation and vitamin B12 and B6 therapy. These interventions have shown promise in lowering homocysteine levels and improving renal function in patients with  DN.

This study focused on the effects of the Tangshen Formula on Hhcy induced by hyper-methionine fed STZ rats, as well as its potential therapeutic role in DN. By comparing the gene methylation patterns between the HM + STZ group and the control group, it was observed that a total of 797 genes exhibited up-regulated methylation status due to HM, which were subsequently down-regulated after TSF treatment. Further analysis revealed 12 genes that were initially hypo-methylated in the HM + STZ group became hyper-methylated after TSF treatment. These findings suggest the potential therapeutic effects of Tangshen Formula in treating DN. Additionally, a comparison with published literature [33–37] identified 19 matched genes among the 797 hyper-to-hypo-methylated genes that are known to be associated with DN, including genes related to oxidative stress, inflammation, and fibrosis. For instance, the SPT20 homolog is a protein encoded by the *SUPT20H* gene in humans and is necessary for the activation of MAP kinase p38 during the process of gastrulation [38]. The *CARD10* gene in humans encodes Caspase recruitment domain-containing protein 10, which activates NF-kappa-B through BCL10 and IKK [39, 40]. In comparison with the control group, our findings indicate that the DN rats exhibited hypo-methylation of the Interleukin-17 receptor B (il17rb) and TNF alpha induced protein 3 (tnfaip3) promoters, as well as low methylation in distal region of the Interleukin-17C (il17c) promoter (data not shown). IL-17 is currently considered a crucial element in the human immune system’s defense mechanism, capable of influencing various types of cells further downstream. Although no evidence of reduced methylation has been discovered in the promoter region of IL-1β, IL-17 has the ability to stimulate macrophages, leading to the secretion of inflammatory substances such as IL-1, tumor necrosis factor (TNF), and IL-6, thereby causing pro-inflammatory effects.

In addition, the study discovered previously unreported genes that could offer valuable insights into the underlying cause of DN and serves as potential targets for further research on therapeutic interventions. One example, KcnJ8, which encodes an ATP-sensitive potassium channel (K-ATP) [41], is widely expressed in different tissues and has been linked to cardiac disorders, specifically early repolarization syndrome (ERS) and Brugada Syndrome (BrS) [42]. Gstt2 is a member of the glutathione S-transferase (GST) family, which is important for drug-metabolizing and has a role in detoxification of xenobiotics and oxidative reactions [43, 44].

Overall, the results of this research indicate that hyper-methionine causes aberrant DNA methylation. Additionally, the Tangshen Formula demonstrates the potential to successfully reverse this disruption, presenting a hopeful treatment choice for DN. The RRBS analysis revealed significant changes in gene methylation patterns in response to Tangshen Formula treatment, highlighting its therapeutic effectiveness. The identification of specific genes associated with DN provides valuable insights into the underlying mechanisms of DN pathogenesis and offers potential therapeutic targets for further investigation.

## Data Availability

The datasets utilized and examined in the present study can be obtained from the corresponding author upon a reasonable request.
